# Inhibition of Larval Development of Marine Copepods *Acartia tonsa* by Neonicotinoids

**DOI:** 10.3390/toxics10040158

**Published:** 2022-03-26

**Authors:** Marco Picone, Gabriele Giuseppe Distefano, Davide Marchetto, Martina Russo, Marco Baccichet, Roberta Zangrando, Andrea Gambaro, Annamaria Volpi Ghirardini

**Affiliations:** 1 Department of Environmental Sciences, Informatics and Statistics, Ca’ Foscari University, Campus Scientifico Via Torino 155, I-30170 Venezia-Mestre, Italy; marco.picone@unive.it (M.P.); gabriele.distefano@unive.it (G.G.D.); martina.russo@unive.it (M.R.); marco.baccichet@unive.it (M.B.); gambaro@unive.it (A.G.); voghi@unive.it (A.V.G.); 2 Institute of Polar Sciences—National Research Council (ISP-CNR), Campus Scientifico Via Torino 155, I-30170 Venezia-Mestre, Italy; rozangra@unive.it

**Keywords:** neonicotinoids, copepods, *Acartia tonsa*, larval development, early-life stages

## Abstract

Neonicotinoids (NEOs) are neurotoxic pesticides widely used in agriculture due to their high effectiveness against pest insects. Despite their widespread use, very little is known about their toxicity towards marine organisms, including sensitive and ecologically relevant taxa such as copepods. Thus, we investigated the toxicity of five widely used NEOs, including acetamiprid (ACE), clothianidin (CLO), imidacloprid (IMI), thiacloprid (THI), and thiamethoxam (TMX), to assess their ability to inhibit the larval development of the copepod *Acartia tonsa*. The more toxic NEOs were ACE (EC_50_ = 0.73 μg L^−1^), TMX (EC_50_ = 1.71 μg L^−1^) and CLO (EC_50_ = 1.90 μg L^−1^), while the less toxic compound was IMI (EC_50_ = 8.84 μg L^−1^). Early life-stage mortality was unaffected by NEOs at all of the tested concentrations. The calculated toxicity data indicated that significant effects due to ACE (EC_20_ = 0.12 μg L^−1^), THI (EC_20_ = 0.88 μg L^−1^) and TMX (EC_20_ = 0.18 μg L^−1^) are observed at concentrations lower than established chronic aquatic life benchmarks reported by USEPA for freshwater invertebrates. Nevertheless, since environmental concentrations of NEOs are generally lower than the threshold concentrations we calculated for *A. tonsa*, the effects may be currently of concern only in estuaries receiving wastewater discharges or experiencing intense runoff from agriculture.

## 1. Introduction

Neonicotinoids (NEOs) are neurotoxic pesticides that disrupt synaptic transmissions by binding with the nicotinic acetylcholine receptors (nAChRs), leading to membrane depolarization, ion channels activation, and propagation of the action potential. In particular, NEOs have a high affinity for nAChRs located in the insects’ central nervous system, and once they bond with nAChRs, they cause neuronal hyper-excitation that produces different sub-lethal effects and also leads to death [1,2]. In addition, some of them (i.e., thiamethoxam) are also suspected to bind to mixed nicotinic/muscarinic receptors [3].

Their high effectiveness against pest insects, combined with high water solubility and low mammalian toxicity, favored their widespread use in agriculture and made them the most popular class of pesticide in the past decades [3,4]. Resistance to hydrolysis and biological degradation confers their environmental persistence, while high water solubility and low soil adsorption facilitate their transport into aquatic systems through runoff and drainage from agricultural land [5,6]. 

Consequently, NEOs have been frequently detected in surface waters worldwide, raising concern for the risk they may pose for non-target aquatic and terrestrial communities supported by these ecosystems [5,7,8]. 

Several studies explored the toxic effects of NEOs on freshwater non-target invertebrates [9,10], and relevant reviews are available too [5,11]. At the same time, data on marine and estuarine species are less copious despite the increasing evidence of their occurrence at detectable concentrations in estuaries and coastal waters [12,13,14,15]. 

Crustaceans are the marine invertebrates most possibly susceptible to NEO’s toxic action due to their nervous system’s similarity with insects [16,17]. Planktonic copepods represent a major component of the marine zooplankton and occupy a critical role in brackish and marine food webs due to their role as grazers on phytoplankton and protozoans and food reservoir for fish larvae [18]. For this reason, toxic effects on copepods may generate disruption of food webs and detrimental effects on higher trophic levels. Many species of copepods are used as bioindicators to assess the adverse effects of chemicals and effluents in surface waters, including *Acartia tonsa*, *Centropages* sp., *Eurytemora affinis* and *Nitocra spinipes* [19,20,21]. In particular, planktonic copepods such as *Acartia tonsa* are very sensitive to pesticides and other organic chemicals acting as endocrine and nervous transmission disruptors [22,23,24,25]. Moreover, the worldwide distribution, easy culturing, short generation times and ecological relevance make *A. tonsa* a useful bioindicator organism for assessing the effects of toxic substances [26].

The present study aimed to provide a first screening of the toxicity of five synthetic commercially available NEO pesticides on the larval development of marine planktonic copepods. The selected active compounds are the first generation NEOs acetamiprid (ACE), imidacloprid (IMI) and thiacloprid (THI), and the second generation NEOs clothianidin (CLO) and thiamethoxam (TMX) [27]. In particular, fertilised eggs of *A. tonsa* were exposed to four concentrations of each pesticide and let to develop for five days to assess the effects of the pesticides on larval development. At the end of the five days exposure, point-estimate toxicity data (EC_10_s, EC_20_s, and EC_50_s) were generated for each pesticide [28,29]. These data were then compared with available aquatic life benchmarks and concentrations in surface waters of estuaries and coastal areas to estimate whether NEOs may pose a risk for marine copepods.

## 2. Materials and Methods

### 2.1. Acartia Tonsa Culturing

Culturing of *A. tonsa* was performed as outlined in Picone et al. [25]. Briefly, adult specimens of *A. tonsa* were purchased from Guernsey Sea Farms Ltd., Port Vale, Guernsey, UK. In-house laboratory cultures were started by adding 800–900 freshly released eggs to 1.8-L of a 20‰ salinity culture medium prepared according to ISO 16778 [30]. The cultures were kept at 20 ± 1 °C in a climatic chamber with a 16-h light and 8-h dark photoperiod and under continuous aeration. The food, consisting of a mixture of three marine flagellates (*Tetraselmis suecica, Pavlova lutheri* and *Tisochrysis lutea*), was provided four times per day through a timer-controlled peristaltic pump. All algal clones were cultured in Guillard’s F/2 medium, at 20 ± 1 °C, under continuous aeration and 16:8 light:dark photoperiod.

The eggs were removed daily from cultures by siphoning off the medium from the bottom of the culture flask and then filtering it through two sieves with mesh sizes of 170-μm and 50-μm, respectively. Adult copepods, retained by the 170-μm mesh sieve, are then reintroduced in the culture, while eggs and nauplii, passing through the 170-μm sieve but retained by the 50-μm sieve, were collected and stored separately. Each culture was maintained for testing for up to 6 weeks. 

Different parental groups (i.e., different *Acartia* cultures) were used during the testing period for a total of five experiments. Eggs from culture AT14/19 were used for testing IMI (14 October 2019), while ACE was tested using eggs from culture AT17/20 (29 June 2020); culture AT18/20 was used for testing TMX (6 July 2020) and THI (13 July 2020), while eggs from culture AT19/20 were used for testing CLO (10 August 2020).

### 2.2. Chemicals

All active NEO compounds were purchased from Merck Life Science s.r.l., Milan, Italy (Supelco PESTANAL, analytical standards). The physicochemical properties of the pesticides are reported in Table 1.

Stock solutions at 10 mg L^−1^ were prepared for each compound in 99% ethanol and then diluted to test concentrations using the 20‰ salinity medium used for culturing the copepods. The solvent percentages in the different NEO tested concentrations were included in the range 0.0001–0.1%. Possible solvent-induced effects were tested by exposing eggs to a series of ethanol concentrations (0.0002–2%) diluted in the 20‰ salinity medium. Ethanol did not significantly affect the larval development of *A. tonsa* at the tested concentrations (0.0002–2%) (F = 1.837; *p* = 0.156). At 0.2% and 2% we observed a minor reduction of LDR as compared with control (14% and 22% inhibition, respectively) but it was not statistically significant (Dunnett post-hoc t-test: *p* = 0.348 and *p* = 0.065, respectively) [25].

Four NEO concentrations were tested for each compound (0.02, 0.21, 2.30 and 21.4 μg L^−1^ for ACE; 0.02, 0.08, 1.32 and 12.4 μg L^−1^ for CLO; 0.02, 0.14, 1.01 and 10.1 μg L^−1^ for IMI; 0.03, 0.14, 1.13 and 11.0 for THI; 0.01, 0.16, 1.01 and 11.0 for TMX). These concentrations bracket the effect-concentrations reported in the literature for acute and early-life stage tests with marine and estuarine crustaceans [5,9,13,31].

### 2.3. Toxicity Testing

The larval development test with *A. tonsa* was performed according to the procedure reported in Picone et al. [25]. Briefly, the test started on day-0 by adding a known number of newly released eggs (up to 80) to a 100 mL glass beaker containing 30 mL of testing solution. Six replicates per NEO concentration and twelve for the negative control were used. The 20‰ salinity culture medium was used as a negative control.

Test vessels were then maintained for five days in a thermostatic incubator (FOC 215E, Velp Scientifica, Milan, Italy) at 20 ± 1 °C, with a 16-h light 8-h dark photoperiod and under a LED illumination to minimize the ultraviolet (UV) emission and avoid photolysis. On day-2, an additional 30 mL of test solution was added to each beaker to refresh the medium. Larvae were fed on day-0 and day-2 with 100 µL of a concentrated (>6 × 10^4^ cell mL^−1^) mixture of *T. suecica*, *T. lutea*, and *P. lutheri* obtained by centrifuging cultured algae per 5 min at 4000× *g*.

Exposures ended on day-5 when approximately 40% of the larvae in negative controls reached the copepodite-I stage. The ratio of nauplii to copepodites was first determined in one control replicate after exactly 5-d by staining the beaker’s content with 0.5 mL of Lugol’s solution (100 g L^−1^ KI, 50 g L^−1^ I_2_, 100 g L^−1^ trichloroacetic acid). Lugol’s solution kills, stains, and preserves unhatched eggs, nauplii, and copepodites [32]. The test solution was then filtered through a mixed cellulose ester filter with gridlines (diameter 47-mm, porosity 0.45-μm), and all of the larvae and unhatched eggs were counted under a dissecting microscope (Stemi SV 6, Zeiss). If the first control contains 40% or more copepodites, the test was finished and also the content of the other beakers was fixed by adding 0.5 mL of Lugol’s solution. Otherwise, the test was run for one additional hour before another control was sacrificed. All unhatched eggs, nauplii and copepodites recovered on the mixed cellulose ester filter were counted under a dissecting microscope to calculate the early-life stages mortality (ELS-m) and the larval development ratio (LDR).

Dissolved oxygen (DO) and pH were measured on day-0 in one beaker per concentration, before the inoculation of the eggs, and on day-5, before staining with Lugol’s solution. 

### 2.4. Endpoints Measured

ELS-m represents the ratio of hatched larvae that die within the fifth day of exposure, and it was calculated as follows:

(1) $${\mathrm{ELS}}_{m}=\frac{\mathrm{initial}\;\mathrm{eggs}-\left(\mathrm{unhatched}\;\mathrm{eggs}+\mathrm{nauplii}+\mathrm{copepodites}\right)\;}{\mathrm{initial}\;\mathrm{eggs}-\mathrm{unhatched}\;\mathrm{eggs}}$$

LDR is the ratio between copepodite-I larvae and the total number of early stages (nauplii plus copepodite-I larvae) recovered at the end of the test:

(2) $$\mathrm{LDR}=\frac{\mathrm{copepodites}}{\mathrm{nauplii}+\mathrm{copepodites}}$$

LDR values obtained for each test concentration were then normalized to the average control LDR to compare results obtained in different testing sessions. Standard error was used as a measure of data dispersion.

### 2.5. Chemical Analysis

NEO testing concentrations were measured using the HPLC/(-)ESI-MS/MS analytical technique, using an Agilent 1100 series HPLC system (Agilent, Waldbronn, Germany) coupled to an API 4000 triple quadrupole mass spectrometer (Applied Biosystems/MDS SCIEX, Toronto, ON, Canada). Briefly, 100 mL of each test solution were diluted with ultrapure water to a final volume of 1 L and spiked with 10 ng of the corresponding deuterated internal standard. The ultrapure water (18.2 MΩcm, 0.01 TOC) was produced by a Chorus system (Elga, High Wycombe, UK). Samples were cleaned up and pre-concentrated using OASIS HLB cartridges (6 cc, 500 mg of sorbent, Waters Milford, MA, USA) previously conditioned with 10 mL of methanol and equilibrated with formic acid (0.2% *v*/*v*) in water (10 mL). After extraction, the cartridges were dried for 5 min and finally NEOs were eluted with 10 mL of methanol. Eluates were reduced to 200 µL under nitrogen flow at 30 °C (Turbovap II^®^, Caliper Life Science, Hopkinton, MA, USA) and reconstituted with 800 µL of ultrapure water. Linearity ranges, limits of detection (LoDs) and limits of quantification (LoQs) are reported in Supplementary Materials—Table S1.

### 2.6. Data Analysis

Effective concentrations 10, (EC_10_s), effective concentrations 20 (EC_20_s), and effective concentrations 50 (EC_50_s) were calculated using a statistical program for generating point-estimate toxicity data for variables with a continuous response, developed at the Technical University of Denmark [33]. Log-normal distribution of the observed effects at the tested concentrations was assumed.

## 3. Results

### 3.1. Quality Assurance/Quality Control (QA/QC)

Five experiments were performed by using eggs collected from four different *A. tonsa* cultures (AT14/19, AT17/20, AT18/20 and AT19/20). Acceptability criteria for negative controls on day-5 include an average LDR of 0.5 ± 0.2 and an average ELS-m less than 0.3. The average LDR obtained in the five experiments for the controls was 0.45 ± 0.05 (*n* = 5), with a minimum of 0.36 ± 0.02 (culture AT17/20, 29 June 2020) and a maximum of 0.64 ± 0.01 (culture AT14/19). ELS-m was less than 0.3 in all tests, with a minimum value of 0.13 ± 0.04 (culture AT14/19) and a maximum of 0.26 ± 0.06 (AT/18, 13 July 2020). All data concerning LDR and ELS-m obtained in negative controls and NEO treatments are reported in Supplementary Materials (Tables S2 and S3).

Positive control tests with 3,5-dichlorophenol (3,5-DCP) as a reference toxicant were used to verify the relative sensitivity of the eggs used in toxicity tests with NEOs, as well as the precision and reliability of the data produced by the laboratory. The control chart acceptability interval for the EC_50_ is 31–250 μg L^−1^ of 3,5-DCP [25,26]. EC_50_s obtained for 3,5-DCP in the four positive control tests performed with the different parental groups (71, 49, 44 and 159 μg L^−1^) were within the control chart’s acceptability interval (31–250 μg L^−1^). Oxygen saturation was always >90% at the beginning and end of the test; pH variation was always < 0.5 units. 

### 3.2. Toxicity Testing

ELS-m was the less sensitive endpoint: none of the tested treatments provided significant mortality compared to negative control. A summary of ELS-m data is reported in Supplementary Materials, Table S2.

In contrast, LDR was heavily affected by all five NEOs (Table 2). According to the calculated EC_50_s, the most toxic NEO pesticide toward *A. tonsa* larval development was ACE, with an EC_50_ of 0.73 μg L^−1^, while the least toxic NEO was IMI (EC_50_ = 8.84 μg L^−1^). The overall toxicity gradient based on EC_50_ was ACE > TMX = CLO > THI > IMI. This gradient was similar to the EC_20_s, with a toxicity gradient of ACE = TMX > CLO > THI > IMI.

**Figure 1:**
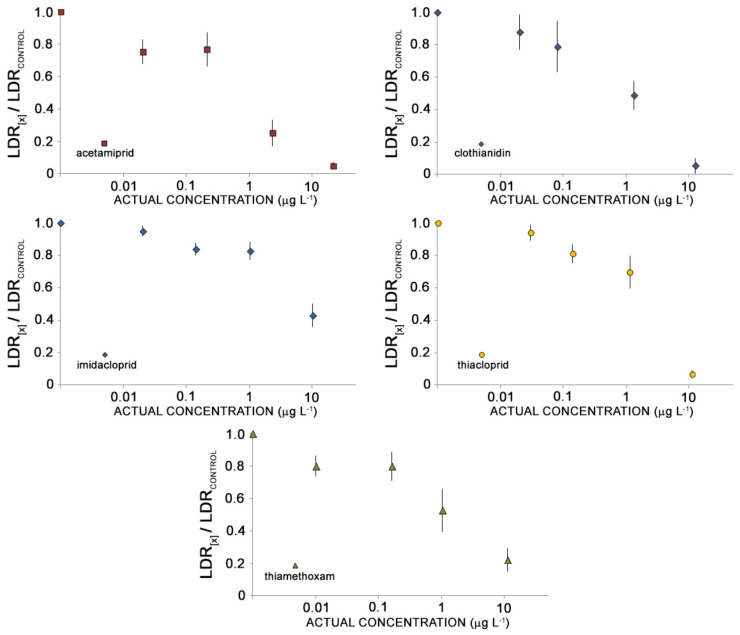
Concentration-effect curve for the tested NEOs. Larval development ratios (LDR_x_) are reported as value normalized to negative control (LDR_control_). Asterisks indicate treatment statistically different from negative control after one-way ANOVA and Dunnett’s post-hoc test (*p* < 0.05).

The effect-concentration curves differed considerably among pesticides (Figure 1). None of the treatments differed significantly from negative control at the lowest tested concentrations, and significant effects were observed only for IMI starting from 0.14 μg L^−1^. At concentrations approximating 1.00 μg L^−1^, toxic effects increased markedly for all pesticides, but IMI, TMX and CLO provided an inhibition of the larval development averaging 50% at 1.01 μg L^−1^ and 1.32 μg L^−1^, respectively, while ACE increased its inhibiting effect on larval development up to 75% at 2.30 μg L^−1^. The toxicity of THI increased moderately, following the log-linear trend exhibited at the lowest tested concentrations (30% inhibition of LDR at 1.13 μg L^−1^).

THI, CLO, and ACE inhibited almost completely the larval development of *A. tonsa* (over 90%) at 11.0, 12.4 and 21.4 μg L^−1^, respectively. TMX and IMI were less toxic than the other pesticides at the highest tested concentrations, with inhibition reaching a maximum of 78% for TMX and 58% for IMI.

## 4. Discussion

### 4.1. The Sensitivity of A. tonsa towards NEOs

The toxicity data reported in the present paper were obtained by using four concentrations spaced by a factor 10; consequently, although the calculated EC_10_s, EC_20_s and EC_50_s are still a good proxy for the assessment of the toxicity of the tested NEOs towards *A. tonsa*, these data may be not appropriate for the derivation of environmental quality criteria. However, the NEO toxicity data showed that the larval development of *A. tonsa* was inhibited at lower concentrations than most of the acute and chronic effect-concentrations reported in the literature for marine crustaceans, including also brackish water copepods, mysids, and prawns (Table 3).

Literature data on the effects of NEOs toward saltwater copepods are available only for the benthic, brackish species *N. spinipes*. Acute effects on adult survival and mobility were observed at a concentration higher than the effective concentration calculated for *A. tonsa*, with 96h-EC_50_s in the range 6.9–120 μg L^−1^ [21]. The larval development test with *N. spinipes* was more sensitive than the acute test and produced 7d-NOECs in the range of 2.5–4.2 μg L^−1^ for THI, IMI and CLO and above 99 μg L^−1^ for TMX [21]. 

As concern other orders, mysids were the marine crustaceans more often used for testing NEOs [5,13]: significant mortality of *Americamysis bahia* (previously *Mysidopsis bahia*) was observed at concentrations ranging from 24 μg L^−1^ (96h-LC_50_ for ACE) up to 4100 μg L^−1^ (96h-LC_50_ for TMX) [15]. Sub-lethal effects on the same species provided 96h-EC_50_s in the range 19–4100 μg L^−1^ [15]. Chronic exposures to NEOs affected *A. bahia* at concentrations significantly lower than 96h-EC/LC_50_s calculated for acute exposure test [9]; as an example, the 28d-NOEC for TMX ranged from 560 μg L^−1^ (survival) to 3600 μg L^−1^ (growth), while the 28d-EC_50_ calculated for ACE was 4.7 μg L^−1^ (growth).

Hano et al. [15] also explored acute effects of NEOs in predominant crustacean species of Japanese estuaries: the reported 96h-EC_50_s ranged from 14 μg L^−1^ (ACE) to 940 μg L^−1^ (TMX) for *Penaeus japonicus*, and 260 μg L^−1^ (CLO) to 3500 μg L^−1^ (ACE) for *Crangon uritai*. All of these data are at least one order of magnitude higher than the EC_50_s calculated for *A. tonsa* LDR in the current study, ranging from 0.73 μg L^−1^ (ACE) to 8.84 μg L^−1^ (IMI). Butcherine et al. [34] evaluated the acute effects of ACE, CLO, IMI and TMX towards *P. monodon* postlarvae, and obtained 48h-LC_50_s values ranging from 190 μg L^−1^ (CLO) up to >500 μg L^−1^ (ACE). However, the authors also observed an increased antioxidant activity at concentrations as low as 5 μg L^−1^.

Data on other marine crustacean species are available only for IMI; only 24h-LC_50_s for juveniles (1.1 mg L^−1^) and megalopae (10 μg L^−1^) of the blue crab *Callinectes sapidus* showed similar or even higher sensitivity as the early-life stages of *A. tonsa* [35]. Other marine species, such as the giant tiger prawn *Penaeus monodon* (48h-LC_50_ = 175 μg L^−1^) and the brine shrimp *Artemia* sp. (48h-LC_50_ > 1.000 μg L^−1^), were by far more tolerant [31,36].

As compared with freshwater taxa, our data for *A. tonsa* are comparable or even lower than effect-concentrations calculated for the most sensitive freshwater species, including Diptera (*Aedes* sp. and *Chironomus dilutus*) and Ephemeroptera (such as *Caenis* sp., *Cloeon* sp., *Neocloen triangulifer*, *Hexagenia* sp.) (Table 4). As an example, Raby et al. [10] reported 96h-EC_50_s ranging from 0.8 μg L^−1^ (THI) to 36.8 μg L^−1^ (TMX) for the midge *Chironomus dilutus*, and from 1.6 μg L^−1^ (ACE) to 5.5 μg L^−1^ (TMX) for the mayfly *Neocloen triangulifer*.

Based on the EC_50_s we calculated in this study (Table 2), NEOs stand among the most effective *A. tonsa* larval development inhibitors. Only the antifouling active ingredient TBT (8d-EC_50_ = 0.003 μg L^−1^) and the fragrance materials amyl-salicylate (5d-EC_50_ = 0.13 μg L^−1^) and hexyl-salicylate (5d-EC_50_ = 0.06 μg L^−1^) provided EC_50_s lower than NEOs [25,40]. Other persistent, bioaccumulative and toxic chemicals such as brominated flame retardants (BDE-28, BDE-47, BDE-99, BDE-100), pesticides (p,p′-DDE) and octyl-phenols (4OP), and some fragrances such as benzyl-salicylate and orange crystals provided 5d-EC_50_s similar to those calculated for the NEO pesticides [24,25,41]. 

**Figure 2:**
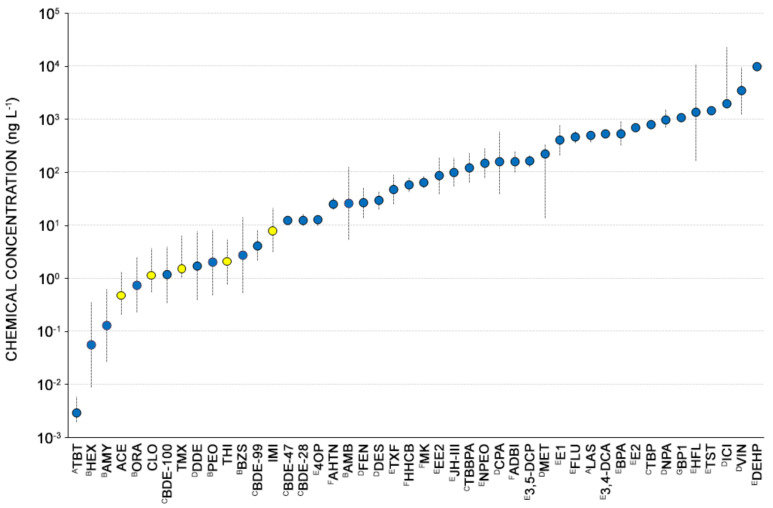
Toxicity of organic micropollutants toward *Acartia tonsa* larval development. All data refers to a 5 d exposure at 20 ± 2 °C; only for TBT and LAS the exposure was prolonged up to 8 d. The 5d-EC_50_s obtained in the present work are highlighted with yellow dots. TBT = tributyltin; HEX = hexil salicylate; AMY = amyl salicylate; ACE = acetamiprid; ORA = oranger crystals; CLO = clothianidin; BDE-100 = 2,2′,4,4′,6-pentabromodiphenyl ether; TMX = thiamethoxam; DDE = p,p′-DDE; PEO = peonile; THI = thiacloprid; BZS = benzyl salicylate; BDE-99 = 2,2′,4,4′,5-pentabromodiphenyl ether; IMI = imidacloprid; BDE-47 = DE-47 = 2,2′,4,4′-tetrabromodiphenyl ether; BDE-28 = 2,4,4′-tribromodiphenyl ether; 4OP = 4-octylphenol; AHTN = Tonalide™; AMB = ambrofix; FEN = fenoxycarb; DES = diethylstilbestrol; TXF = tamoxifen; HHCB = Galaxolide™; MK = musk ketone; EE2 = 17α ethinylestradiol; JH-III = juvenile hormone III; TBBPA = tetrabromobisphenol A; NPEO = nonylphenol ethoxylate; CPA = cyproterone acetate; ADBI = Celestolide™; 3,5-DCP = 3,5-dichlorophenol; MET = methoprene; E1 = estrone; FLU = flutamide; LAS = linear alkylbenzene sulfonate; DCA = 3,4-dichloroaniline; BPA = bisphenol A; E2 = 17β estradiol; TBP = 2,4,6-tribromophenol; NPA = nonylphenol acetate; BP1 = 2,4-dihydroxybenzophenone; HFL = hydroxyflutamide; TST = testosterone; ICI = ICI 182780; VIN = vinclozin; DEHP = diethyl phthalate. ^A^ [40]; ^B^ [25]; ^C^ [24]; ^D^ [41]; ^E^ [43]; ^F^ [23]; ^G^ [42].

In contrast, estrogens (E1, E2 and EE2) [32] and other possible endocrine-disrupting chemicals including pharmaceuticals (flutamide, tamoxifen, hydroxyflutamide) [32], synthetic musks (Tonalide™, Galaxolide™, Celestolide™, musk ketone) [23], phtalates (DEHP) [32], pesticides (methoprene, fenoxycarb and vinclozolin) [41], ultraviolet filters (BP1) [42] and other fragrance materials (ambrofix, peonile) [25] provided 5d-EC_50_ values ranging from 490 μg L^−1^ (Tonalide™) [23] to 1400 μg L^−1^ (DEHP) [32], markedly higher than the 5d-EC_50_ obtained for NEOs in the present study (Figure 2).

### 4.2. Comparative Toxicity of NEOs

The effect-concentration data obtained with *A. tonsa* evidenced a different sensitivity toward the different NEOs, with ACE (EC_50_ = 0.73 μg L^−1^) characterized by a higher inhibitory potential than the other NEOs. A different tolerance towards NEO compounds is a typical output for several species, sensitive freshwater insects (*C. dilutus*, *N. triangulifer*), oligochaetes (*Lumbriculus variegatus*) and both freshwater (*Hyalella azteca*, *Gammarus pulex*) and marine crustaceans (*A. bahia*, *P. japonicus* and *N. spinipes*) [5,10,15,21]. 

The binding properties at the nicotinic cholinergic receptors (nAChR) may explain the different species-specific sensitivities towards NEOs. Recent studies on chironomids (*C. riparius* and *C. dilutus*) evidenced that factors such as nAChR density, receptor binding affinity and compound-specific binding affinity may be responsible for species-specific responses amongst different species and life-stages [44]. According to these findings, the lower toxicity of IMI we observed toward *A. tonsa* compared with ACE and CLO could be defined by an IMI lower binding affinity to the nAChR of the naupliar stage compared with the other NEOs, similarly to that observed for chironomids [45]. 

### 4.3. Environmental Significance of NEOs Toxicity toward Copepods

The effect-concentrations calculated for *A. tonsa* are generally lower than the acute aquatic life benchmarks for freshwater invertebrates proposed by USEPA, and in several cases, also below the chronic aquatic life benchmarks (CALBs). In particular, EC_10_s and EC_20_s calculated for *A. tonsa* are equal or below the CALBs for ACE, THI and TMX, as reported in Table 2. In contrast, as concern IMI, calculated EC_10_ and EC_20_ are higher than the USEPA CALBs and the standards proposed for long-term exposures by European Commission (8.3 ng L^−1^) [45]. These data underline that the existing legislation may have, up to now, underestimated NEOs’ possible long-term effects in marine invertebrates, especially for ACE, THI and TMX. 

On the other hand, the EC_10_s we obtained for *A. tonsa* larval development for CLO, IMI, THI and TMX are higher than the annual average environmental quality standards (AA-EQS) recently proposed for saltwater (Table 2). At the same time, only for TMX the calculated EC_20_ is lower than the proposed maximum allowable concentration environmental quality standards (MAC-EQS) for saltwater [21]. Based on these data, the saltwater EQS proposed by Moeris et al. [21] seem more appropriate than previously available benchmarks to assess the risk posed by NEOs pesticides in estuaries and coastal waters.

Moreover, the available literature data attest that NEOs in brackish and coastal waters generally occur at concentrations at least one order of magnitude lower than the effect-concentrations calculated for LDR in *A. tonsa* and the benchmark proposed for preserving aquatic invertebrates. For example, in Jiaozhou Bay, China, only IMI and ACE were detected, but their concentration was <1 ng L^−1^ in all of the sampling stations [46]; similarly, monitoring of pesticides in inshore waters of the Great Barrier Reef (Australia) reported a maximum concentration of IMI of 1.6 ng L^−1^ [12]. Monitoring of pesticides in estuarine and coastal areas finally confirms that NEOs’ inputs may exceed established benchmarks in presence of seasonal runoff from agricultural land or point discharges. In River Colne, UK, ACE and IMI concentrations downstream from the discharge point of a wastewater treatment plant exceeded EC_10_s for both pesticides and EC_20_ for ACE (0.19–0.29 and 0.06–0.17 μg L^−1^, respectively for ACE and IMI) [47]. Conversely, in the Seto Inland Sea (Japan), maximum detected IMI concentrations (0.213 µg L^−1^) approached the EC_10_ calculated for *A. tonsa* only during intense application in agricultural land, from June to September, while in other seasons, pesticide concentrations were lower [15].

## 5. Conclusions

The calculated effect-concentration data indicated that NEOs are potent inhibitors of larval development. Significant effects due to ACE, THI and TMX on *A. tonsa* were observed at concentrations lower than established chronic aquatic life benchmarks reported by USEPA for freshwater invertebrates. However, effect concentrations calculated for *A. tonsa* larval development are higher than the recently proposed EQS for saltwater, and environmental concentrations in estuarine and coastal areas seldom exceed these benchmarks and effect-concentrations calculated for *A. tonsa*. Based on the actual contamination levels, larval development of copepods might be impaired only in estuaries receiving wastewater treatment plant’s discharges or intense runoff from agricultural land during the season of pesticide’s application.

## Figures and Tables

**Table 1:** Chemical properties of the tested NEO pesticides.

	CAS Number	Chemical Formula	Molar Mass (g mol^−1^) ^†^	Water Solubility at 20 °C (mg L^−1^) ^†^	Vapor Pressure at 20 °C (mPa) ^†^	Log K_ow_^†^	Photolysis (t_1/2_ in d) ^†^	Hydrolysis (t_1/2_ in d) ^†^
acetamiprid	135410-20-7	C_10_H_11_ClN_4_	222.7	2950	1.7 × 10^−4^	0.80	34	stable
clothianidin	210880-92-5	C_6_H_8_ClN_5_O_2_S	249.7	340	2.8 × 10^−8^	0.91	<1	stable
imidacloprid	138261-41-3	C_9_H_10_ClN_5_O_2_	255.7	610	4.0 × 10^−7^	0.57	<1	stable
thiacloprid	111988-49-9	C_10_H_9_ClN_4_S	252.7	184	3.0 × 10^−7^	1.26	stable	stable
thiamethoxam	153719-23-4	C_8_H_10_ClN_5_O_3_S	291.7	4100	6.6 × 10^−6^	−0.13	2.7	stable

^†^ Data from Pesticide Properties DataBase (PPDB), University of Hertfordshire. http://sitem.herts.ac.uk/aeru/ppdb/en/index.htm (accessed on 14 March 2022).

**Table 2:** Summary of the effect concentrations calculated for the NEOs. Data are expressed in μg L^−1^. US Environmental Protection Agency (USEPA) Office of Pesticide Programs (OPP) aquatic life benchmarks were retrieved from https://www.epa.gov/pesticide-science-and-assessing-pesticide-risks/aquatic-life-benchmarks-and-ecological-risk, accessed on 2 June 2021. Environmental quality standards for saltwater were retrieved from Moeris et al. [21].

	Effect-Concentrations for *A. tonsa* and 95% Confidence Interval			USEPA Aquatic Life Benchmarks		Environmental Quality Standards for Saltwater ^†^	
	EC_10_	EC_20_	EC_50_	Acute	Chronic	AA-EQS	MAC-EQS
acetamiprid	0.05 (0.01–1.18)	0.12 (0.01–1.75)	0.73 (0.25–2.13)	10.5	2.1	-	-
clothianidin	0.30 (0.04–2.11)	0.56 (0.13–2.41)	1.90 (0.99–3.63)	11	0.05	0.05	0.23
imidacloprid	0.50 (0.11–2.22)	1.33 (0.43–4.11)	8.84 (5.13–15.24)	0.385	0.01	0.002	0.065
thiacloprid	0.53 (0.21–1.34)	0.88 (0.44–1.76)	2.34 (1.49–3.69)	18.9	0.97	0.0048	0.46
thiamethoxam	0.06 (0.01–0.71)	0.18 (0.06–1.25)	1.71 (0.61–4.80)	17.5	0.74	0.016	5.2

^†^ AA-EQS, Annual Average Environmental Quality Standard; MAC-EQS, Maximum Allowable concentration Environmental Quality Standard.

**Table 3:** Toxicity of NEOs towards marine and brackish crustaceans. All data are reported in µg L^−1^. NOEC, no observed effect concentration; LOEC, lowest observed effect concentration.

Species	Endpoint	Parameter	ACE	CLO	IMI	THI	TMX	Reference
*Nitocra spinipes*	Mortality	96h-EC_50_	-	6.9	25.0	7.2	120	[21]
	Larval development	7d-NOEC	-	2.5	4.2	2.7	>99	
*Americamysis bahia*	Mortality	96h-LC_50_	24.0	51.0	160	67.0	4100	[15]
	Immobility	96h-EC_50_	19.0	48.0	92.0	47.0	4100	
	Mortality	96h-LC_50_	-	-	-	-	6900	[9]
	Survival	28d-NOEC	-	-	-	-	560	
		28d-LOEC	-	-	-	-	1100	
	Growth	28d-NOEC	-	-	-	-	3900	
		28d-LOEC	-	-	-	-	>3900	
*Penaeus japonicus*	Mortality	96h-LC_50_	85	89	71	64	3900	[15]
	Immobility	96h-EC_50_	31	14	50	20	940	
*Crangon uritai*	Mortality	96h-LC_50_	4500	360	2200	1800	2200	[15]
	Immobility	96h-EC_50_	3500	260	570	490	820	
*Penaeus monodon*	Mortality (postlarvae)	48h-LC_50_	>500	190	408	-	390	[34]
	Mortality (postlarvae)	48h-LC_10_	-	-	3	-	-	[31]
		48h-LC_50_	-	-	175	-	-	
*Callinectes sapidus*	Mortality (megalopae)	24h-LC_50_	-	-	10	-	-	[35]
	Mortality (juveniles)	24h-LC_50_	-	-	1112	-	-	
*Artemia* sp.	Mortality	48h-LC_50_	-	-	361,230	-	-	[36]

**Table 4:** Toxicity of NEOs towards selected freshwater crustaceans (*Daphnia magna*, *Ceriodaphnia dubia*, *Hyalella azteca*), Ephemeroptera (*Caenis* sp., *Cloeon* sp., *Ephemerella* sp., *Hexagenia* sp., *Isonychia bicolor*, *McCaffertium* sp., *Neocloen triangulifer*), Odonata (*Coenagrion* sp.), Hemiptera (*Trichocorixa* sp.), Tricoptera (*Cheumatopsyche* sp.), and Diptera (*Aedes* sp., *Chironomus dilutes*). All data are reported in µg L^−1^. NOEC = No observed effect concentration; LOEC = Lowest observed effect concentration.

Species	Endpoint	Parameter	ACE	CLO	IMI	THI	TMX	Reference
*Daphnia magna*	Mortality	48h-LC_50_	-	-	>102,000	-	>80,000	[10]
	Mortality	48h-LC_50_	-	-	10,440	-	-	[36]
*Ceriodaphnia dubia*	Mortality	48h-LC_50_	>33,500	>100,000	72125	>41,500	>80,000	[10]
*Hyalella azteca*	Mortality	96h-LC_50_	4.8	5.2	363.2	55	801	[10]
	Immobility	96h-LC_50_	4.4	4.8	176.9	26.9	391	
	Mortality	7d-LC_50_	4.7	4.0	230	68	290	[37]
	Survival	28d-LC_50_	4.2	3.4	90	44	220	
	Growth	28d-EC_50_	3.4	3.5	4.3	42	200	
*Caenis* sp.	Mortality	96h-LC_50_	783	122	<21.8	231	382	[10]
	Immobility	96h-EC_50_	<138.8	-	<21.8	<66.3	<23.3	
*Cloeon* sp.	Mortality	96h-LC_50_	2368	3939	1152	3883	4633	[10]
	Immobility	96h-EC_50_	<16.6	<16.4	23.1	23.1	44.1	
*Ephemerella* sp.	Mortality	96h-LC_50_	158.2	586.9	68.2	190.6	334.9	[10]
	Immobility	96h-EC_50_	<56.1	18.5	10.6	<58	<59	
*Hexagenia* sp.	Mortality	96h-LC_50_	>35,600	>17,400	9321	>9300	>30,800	[10]
	Immobility	96h-EC_50_	1.8	5.5	n.c.	<1.3	35.8	
	Mortality	96h-LC_50_	780	2000	900	6200	>10,000	[37]
	Mortality	96h-NOEC	1	10	1	1	100	
	Behaviour	96h-EC_50_	4.0	24	10	9.1	630	
*Isonychia bicolor*	Mortality	96h-LC_50_	>9600	>1740	715	-	>7120	[10]
	Immobility	96h-EC_50_	<600	<109	60.4	-	<445	
*McCaffertium* sp.	Mortality	96h-LC_50_	>890	1328	1810	>920	>920	[10]
	Immobility	96h-EC_50_	<56.1	<109	10.6	10.6	81.7	
*Neocloen triangulifer*	Mortality	96h-LC_50_	1.7	3.5	5.2	1.9	5.5	[10]
	Immobility	96h-EC_50_	1.6	3.5	3.1	1.9	5.5	
*Coenagrion* sp.	Mortality	96h-LC_50_	24,393	14,556	3463	5647	15,062	[10]
	Immobility	96h-EC_50_	<5625	<5919	<5438	<2500	<4188	
*Trichocorixa* sp.	Mortality	48h-LC_50_	1515	34.8	450.4	135.3	1473	[10]
	Immobility	48h-EC_50_	63.5	21.3	63.1	<39.7	56.3	
*Cheumatopsyche* sp.	Mortality	96h-LC_50_	403.8	1281	324.5	>920	170.1	[10]
	Immobility	96h-EC_50_	<56.1	<108.8	176.4	162.6	118.5	
*Chironomus dilutus*	Mortality	96h-LC_50_	2.8	11.6	11.8	1.6	61.9	[10]
	Immobility	96h-EC_50_	2.7	3.4	2.5	0.8	36.8	
	Mortality	14d-LC_50_	-	2.4	1.5	-	23.6	[38]
	Growth	14d-EC_50_	-	1.8	2.2	-	21.4	
	Emergence	40d-EC_50_	-	0.3	0.4	-	4.1	
	Mortality	96h-LC_50_	-	-	7.0	-	-	[39]
*Aedes* sp.	Mortality	48h-LC_50_	159.6	28.5	40.8	53.4	67.4	[10]
*Aedes aegypti*	Mortality	48h-LC_50_	-	-	44	-	-	[36]
*Aedes taeniorhynchus*	Mortality	48h-LC_50_	-	-	13	-	-	[36]

## Data Availability

The data that supports the findings of this study are available in the paper and in Supplementary Materials of this article.

## Supplementary Materials

The following supporting information can be downloaded at: https://www.mdpi.com/article/10.3390/toxics10040158/s1. Table S1: Linearity ranges, limits of detection (LOD)s, limits of quantification (LOQs) and R2, for the studied NEOs; Table S2: Summary of the larval mortality (ELS-m) observed in the tested treatments. Data are reported as mean ± standard error. Actual concentrations are reported in µg L^−1^; Table S3: Summary of the larval development ratio (LDR) observed in the tested treatments. Actual concentrations are reported in µg L^−1^. Data are reported as mean ± standard error. Significant differences (*p* < 0.05) as compared with control are highlighted in bold.
