# Supplementary material for: Inhibition of Larval Development of Marine Copepods Acartia tonsa by Neonicotinoids
Source: Toxics. 2022 Mar 26;10(4):158. doi: 10.3390/toxics10040158 (PMC9026078; doi:10.3390/toxics10040158)
Supplement: Supplementary file 1 [file toxics-10-00158-s001.zip › toxics-1625513-supplementary.pdf]

toxics

MDPI

Supplementary Materials: Inhibition of Larval Development of Marine Copepods *Acartia tonsa* by Neonocotinoids

Marco Picone, Gabriele Giuseppe Distefano, Davide Marchetto, Martina Russo, Marco Baccichet, Roberta Zangrando, Andrea Gambaro, Annamaria Volpi Ghirardini

Table S1. Linearity ranges, limits of detection (LOD)s, limits of quantification (LOQs) and R^2, for the studied neonicotinoids.

Compound	Linearity range (ng mL^{-1})	LOD (pg mL^{-1})	LOQ (pg mL^{-1})	R^2
ACE	0.008–1000	2	8	0.9997
IMI	0.022–1100	6	22	0.9991
CLO	0.049–600	15	49	0.9857
TCLO	0.005–1050	2	5	0.9935
TMX	0.061–1200	18	61	0.9965

Table S2. Summary of the larval mortality (ELS-m) observed in the tested treatments. Data are reported as mean ± standard error. Actual concentrations are reported in µg L^{-1}.

	Culture	Concentration	ELS-m	One-way ANOVA
ACE	AT17/20	Control	0.26 ± 0.03	$F_{4,28} = 1.81$, $p = 0.156$
		0.02	0.22 ± 0.05	
		0.21	0.31 ± 0.04	
		2.30	0.39 ± 0.06	
		21.4	0.25 ± 0.05	
CLO	AT19/20	Control	0.20 ± 0.01	$F_{4,29} = 2.42$, $p = 0.072$
		0.02	0.11 ± 0.01	
		0.08	0.26 ± 0.01	
		1.32	0.17 ± 0.01	
		12.4	0.27 ± 0.01	
IMI	AT14/19	Control	0.13 ± 0.04	$F_{4,31} = 0.97$, $p = 0.439$
		0.02	0.28 ± 0.13	
		0.14	0.18 ± 0.04	
		1.01	0.25 ± 0.08	
		10.1	0.13 ± 0.06	
THI	AT18/20	Control	0.26 ± 0.06	$F_{4,28} = 1.48$, $p = 0.239$
		0.03	0.45 ± 0.10	
		0.14	0.19 ± 0.03	
		1.13	0.17 ± 0.06	
		11.0	0.26 ± 0.09	
TMX	AT18/20	Control	0.17 ± 0.07	$F_{4,29} = 2.57$, $p = 0.059$
		0.01	0.32 ± 0.04	
		0.16	0.33 ± 0.06	
		1.01	0.29 ± 0.05	
		11.0	0.26 ± 0.02	

Table S3. Summary of the larval development ratio (LDR) observed in the tested treatments. Actual concentrations are reported in µg L^{-1}. Data are reported as mean ± standard error. Significant differences ($p < 0.05$) as compared with control are highlighted in bold.

	**Culture**	**Concentration**	**LDR**	**One-way ANOVA**	**Dunnet's *post-hoc* test**
ACE	AT17/20	Control	0.36 ± 0.02	$F_{4,28} = 31.53$, $p < 0.001$	-
		0.02	0.25 ± 0.01		$p = 0.094$
		0.21	0.28 ± 0.01		$p = 0.271$
		2.30	**0.08 ± 0.01**		**$p < 0.001$**
		21.4	**0.02 ± 0.01**		**$p < 0.001$**
CLO	AT19/20	Control	0.48 ± 0.03	$F_{4,29} = 22.15$, $p < 0.001$	-
		0.02	0.43 ± 0.05		$p = 0.358$
		0.08	0.38 ± 0.08		$p = 0.144$
		1.32	**0.27 ± 0.05**		**$p < 0.001$**
		12.4	**0.03 ± 0.01**		**$p < 0.001$**
IMI	AT14/19	Control	0.64 ± 0.01	$F_{4,31} = 29.20$, $p < 0.001$	-
		0.02	0.61 ± 0.02		$p = 0.485$
		0.14	**0.53 ± 0.02**		**$p = 0.008$**
		1.01	**0.53 ± 0.04**		**$p = 0.017$**
		10.1	**0.27 ± 0.05**		**$p < 0.001$**
THI	AT18/20	Control	0.49 ± 0.04	$F_{4,28} = 13.72$, $p < 0.001$	-
		0.03	0.46 ± 0.02		$p = 0.632$
		0.14	0.40 ± 0.03		$p = 0.167$
		1.13	**0.34 ± 0.06**		**$p = 0.023$**
		11.0	**0.03 ± 0.01**		**$p < 0.001$**
TMX	AT18/20	Control	0.42 ± 0.06	$F_{4,27} = 8.44$, $p < 0.001$	-
		0.01	0.33 ± 0.03		$p = 0.322$
		0.16	0.34 ± 0.04		$p = 0.331$
		1.01	**0.22 ± 0.07**		**$p = 0.005$**
		11.0	**0.09 ± 0.03**		**$p < 0.001$**
